# The genome of *Ensifer alkalisoli* YIC4027 provides insights for host specificity and environmental adaptations

**DOI:** 10.1186/s12864-019-6004-7

**Published:** 2019-08-12

**Authors:** Xiaoxiao Dang, Zhihong Xie, Wei Liu, Yu Sun, Xiaolin Liu, Yongqiang Zhu, Christian Staehelin

**Affiliations:** 1 Key Laboratory of Coastal Biology and Bioresource Utilization, Yantai Institute of Coastal Zone Research, Chinese Academy of Sciences, Yantai, China; 2 University of Chinese Academy of Sciences, Beijing, China; 3 Center for Ocean Mag-Science, Chinese Academy of Sciences, Qingdao, People’s Republic of China; 4 Shanghai-MOST Key Laboratory of Health and Disease Genomics, Chinese National Human Genome Center at Shanghai, Shanghai, 201203 China; 5 State Key Laboratory of Biocontrol and Guangdong Key Laboratory of Plant Resources, School of Life Sciences, Sun Yat-sen University, Guangzhou, 510006 China

**Keywords:** *Ensifer alkalisoli*, Complete genome sequencing, Comparative genomics, Host-specific symbiosis, Environmental adaptation

## Abstract

**Background:**

*Ensifer alkalisoli* YIC4027, a recently characterized nitrogen-fixing bacterium of the genus *Ensifer*, has been isolated from root nodules of the host plant *Sesbania cannabina*. This plant is widely used as green manure and for soil remediation. *E. alkalisoli* YIC4027 can grow in saline-alkaline soils and is a narrow-host-range strain that establishes a symbiotic relationship with *S. cannabina.* The complete genome of this strain was sequenced to better understand the genetic basis of host specificity and adaptation to saline-alkaline soils.

**Results:**

*E. alkalisoli* YIC4027 was found to possess a 6.1-Mb genome consisting of three circular replicons: one chromosome (3.7 Mb), a chromid (1.9 Mb) and a plasmid (0.46 Mb). Genome comparisons showed that strain YIC4027 is phylogenetically related to broad-host-range *Ensifer fredii* strains. Synteny analysis revealed a strong collinearity between chromosomes of *E. alkalisoli* YIC4027 and those of the *E. fredii* NGR234 (3.9 Mb), HH103 (4.3 Mb) and USDA257 (6.48 Mb) strains. Notable differences were found for genes required for biosynthesis of nodulation factors and protein secretion systems, suggesting a role of these genes in host-specific nodulation. In addition, the genome analysis led to the identification of YIC4027 genes that are presumably related to adaptation to saline-alkaline soils, rhizosphere colonization and nodulation competitiveness. Analysis of chemotaxis cluster genes and nodulation tests with constructed *che* gene mutants indicated a role of chemotaxis and flagella-mediated motility in the symbiotic association between YIC4027 and *S. cannabina*.

**Conclusions:**

This study provides a basis for a better understanding of host specific nodulation and of adaptation to a saline-alkaline rhizosphere. This information offers the perspective to prepare optimal *E. alkalisoli* inocula for agriculture use and soil remediation.

**Electronic supplementary material:**

The online version of this article (10.1186/s12864-019-6004-7) contains supplementary material, which is available to authorized users.

## Background

Rhizobia are soil bacteria that can establish a mutualistic symbiosis with leguminous plants by forming nitrogen-fixing nodules. Within the nodules, rhizobia convert atmospheric nitrogen into ammonia, which can then be used as a nitrogen source. Increased nitrogen availability results in improved growth and productivity of host plants [1].

*Sesbania cannabina* is an annual fast-growing semi-shrub belonging to the Leguminosae family. Due to its outstanding resistance to salt and flooding stress, *S. cannabina* is widely cultivated in subtropical and tropical regions of Asia, Africa and Australia for various purposes such as green manure, salinization alleviation, and land remediation [2, 3]. In China, *S. cannabina* has been successfully introduced as a pioneer plant in the barren saline-alkaline land of the Yellow River Delta (YRD) [4]. However, despite the economic and environmental importance of this plant, little is known of its nitrogen-fixing symbionts which can considerably promote plant growth. To obtain more benefits from *S. cannabina*, studies on its microsymbiont are needed.

Previous work on bacteria associated with *S. cannabina* grown in saline-alkaline soil of YRD led to the identification of strains belonging to various genera of the *Rhizobiaceae* family, i.e. *Ensifer* (*Sinorhizobium*), *Rhizobium*, *Neorhizobium* and *Agrobacterium* [5]. Among these bacteria, nitrogen-fixing *Ensifer* strains differing from previously characterized *Ensifer* strains were identified based on multilocus sequence analysis and average nucleotide identity (ANI). These strains were dominant, i.e. accounting for 73% of the local isolates [5]. This led us to propose a new species, *E. alkalisoli*, with YIC4027 as a type strain. *E. alkalisoli* is closely related to *E. fredii* and *E. sojae* [6]. *E. alkalisoli* YIC4027 displays symbiotic nitrogen fixation ability, salt tolerance (up to 4% NaCl) and alkaline tolerance (pH 6–10) [6]. YIC4027 can enter a nodule symbiosis, but its host range is limited and *S. cannabina* is so far the only known host plant of YIC4027. YIC4027 efficiently colonizes the rhizosphere of *S. cannabina* roots, suggesting a high degree of nodulation competitiveness. Compared to other *E. alkalisoli* strains, YIC4027 showed strongest plant growth-promoting effects under greenhouse conditions and in field plot experiments (unpublished data). Thus, YIC4027 can be potentially used as valuable inoculant for *S. cannabina*.

The *Ensifer* genus, which belongs to the alpha subgroup of *Proteobacteria*, is one of the most widely studied group of rhizobia. The alfalfa symbiont *E. meliloti* Rm1021 (formerly *Sinorhizobium meliloti*) was the first completely sequenced *Ensifer* strain [7]. Rm1021 is a classic narrow-host-range strain that can induce nodules only on three legume genera, namely *Medicago*, *Melilotus* and *Trigonella* [8]. In contrast, the *E. fredii* strains NGR234 (isolated from *Lablab purpureus*), USDA257 and HH103 (both isolated from soybean), which are phylogenetically closely related to *E. meliloti* [9], are typical broad-host-range rhizobia that enter symbiosis with hosts belonging to more than 79 different genera of legumes [10, 11]. Host specificity is an intriguing but still poorly understood feature of the nodule symbiosis [9, 12]. Comparative analyses of rhizobial genomes combined with knowledge on the chemical nature of host range determinants can provide useful information on genes involved in host specificity [13].

In the present work, a genome sequence analysis was performed to explore the presence or absence of symbiosis-related *E. alkalisoli* YIC4027 genes. The YIC4027 genome was compared with available genomes of closely related strains (*E. fredii* NGR234, USDA257, and HH103) in order to identify gene candidates that may account for host specific nodulation. The genome analysis also resulted in identification of genes that may help the bacterium to colonize the rhizosphere, i.e. genes possibly related to adaptation to saline-alkaline soils and nodulation competitiveness.

## Results

### General genomic features of *E. alkalisoli* YIC4027

**Fig. 1:**
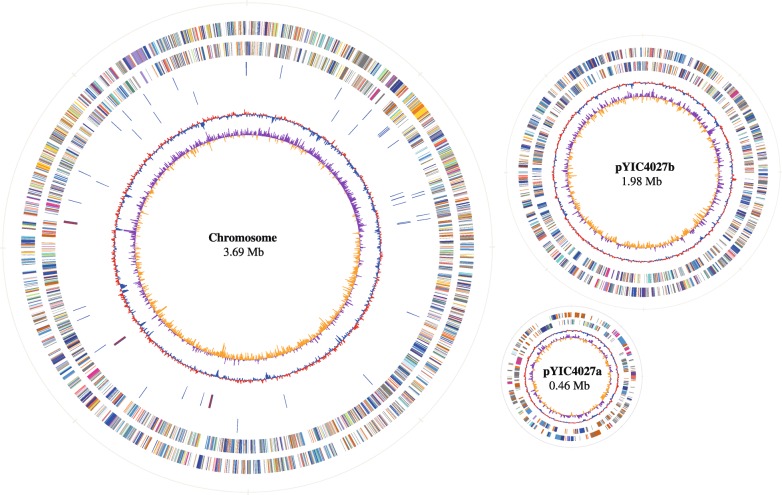
Circular plots of the three replicons of *E. alkalisoli* YIC4027. The outermost and second-outermost circles represent the predicted protein coding sequences on the forward and the reverse strand, respectively. They are colored according to the assigned cluster of orthologous group classes. The innermost and second-innermost circles show the GC skew values and GC content, respectively. GC skew values are colored purple for plus values and yellow for minus values. GC content circles show deviations from the average (projecting outward for values higher than the average and projecting inward for values lower than the average). For the chromosome, the predicted RNA sequences on the forward and the reverse strands are shown in the circles 3 and 4 from the outermost to the innermost circles, respectively. The genes for rRNA are marked in red and those for tRNA in blue. All three replicons are drawn at the same scale

The complete genome of YIC4027 was sequenced using a Pacific Biosciences platform (accession numbers CP034909 to CP034911). Circular genome plots of the replicons are shown in Fig. 1 and their main features are presented in Table 1. The genome consists of 6,128,433 base pairs (bp) and has three circular replicons: one large chromosome of 3,690,234 bp, pYIC4027a, a plasmid of 456,424 bp carrying nodulation genes (referred as the symbiotic plasmid), and pYIC4027b, a chromid of 1,981,775 bp (Fig. 1 and Table 1). The GC content of pYIC4027a is 59.3%, which is lower than that of the chromosome (62.6%) or pYIC4027b (62.3%). This suggests that pYIC4027a could have been acquired by horizontal gene transfer from other bacteria. All RNA genes are located on the chromosome. The three identified rRNA gene clusters were found to be in the order 16S–23S-5S. The 55 tRNA genes representing 43 tRNA species (for 21 amino acids) are scattered throughout the chromosome and are probably transcribed as single units. Coding sequences (CDSs) cover 86.2% of the whole genome. Totally 6024 CDSs were predicted and the average CDS size was 876 bp. Among the CDSs, 4540 (75.4%) genes were annotated as genes with known biological functions, while 1484 (24.6%) encode hypothetical proteins (Table 1).

**Table 1:** General genomic features of *E. alkalisoli* YIC4027

Feature	*E. alkalisoli* YIC4027
Genome size (bp)	6,128,433
Chromosome (bp)	3,690,234
pYIC4027b (bp)	1,981,775
pYIC4027a (bp)	456,424
GC content(%)	62.2
CDS coverage(%)	86.2
CDS number	6024
Number of genes with known function	4540 (75.4%)
Hypothetical proteins	1484 (24.6%)
tRNA genes	55
rRNA operons	3
Average gene length (bp)	876
Genes assigned to COG	4555 (75.6%)
Genes assigned to KEGG	2114 (35.1%)

We predicted gene functions using Clusters of Orthologous Groups of proteins (COG). Among the identified CDSs, 4555 (75.6%) genes were classified into COG families composed of 21 categories (Table 2 and Additional file 1: Figure S1). The results revealed three main functional gene classes: amino acid transport and metabolism, carbohydrate transport and metabolism and transcription, representing 22.9% of the predicted CDS, while 16.6% of the predicted CDS were poorly characterized.

**Table 2:** COG categorization of *E. alkalisoli* YIC4027 CDSs

COG functional categories	CDSs	% of CDSs
Metabolism		
C-Energy production and conversion	298	4.95
E-Amino acid transport and metabolism	557	9.25
F-Nucleotide transport and metabolism	86	1.43
G-Carbohydrate transport and metabolism	438	7.27
H-Coenzyme metabolism	158	2.62
I-Lipid metabolism	139	2.31
P-Inorganic ion transport and metabolism	181	3.00
Q-Secondary metabolites biosynthesis, transport, and catabolism	113	1.88
Cellular processes and signaling		
D-Cell division and chromosome partitioning	32	0.53
M-Cell envelope biogenesis, outer membrane	229	3.80
N-Cell motility and secretion	77	1.28
O-Post-translational modification, protein turnover, chaperones	175	2.91
T-Signal transduction mechanisms	184	3.05
U-Intracellular trafficking and secretion	91	1.51
V-Defense mechanisms	54	0.90
Information storage and processing		
B-Chromatin structure and dynamics	2	0.03
J-Translation, ribosomal structure and biogenesis	169	2.81
K-Transcription	382	6.34
L-DNA replication, recombination, and repair	193	3.20
Pooly characterized		
R-General function prediction only	492	8.17
S-Function unknown	505	8.38
Total	4555	75.61

Furthermore, the predicted genes of YIC4027 were categorized into 20 KEGG (Kyoto Encyclopedia of Genes and Genomes) classes (Table 3 and Additional file 2: Figure S2). Many genes were attributed to three categories, namely amino acid metabolism (4.9%), membrane transport (4.7%) and carbohydrate metabolism (4.5%). These findings confirmed a preference toward metabolism and transport of amino acids and carbohydrates, consistent with the results obtained from COG functional analysis.

**Table 3:** KEGG pathway categorization of *E. alkalisoli* YIC4027 CDSs

KEGG pathway functional categories	CDSs	% of CDSs
Metabolism		
– Carbohydrate metabolism	273	4.53
– Energy metabolism	223	3.70
– Lipid metabolism	92	1.53
– Nucleotide metabolism	112	1.86
– Amino acid metabolism	295	4.90
– Metabolism of other amino acids	87	1.44
– Glycan biosynthesis and metabolism	37	0.61
– Metabolism of cofactors and vitamins	163	2.71
– Metabolism of terpenoids and polyketides	35	0.58
– Biosynthesis of other secondary metabolites	25	0.42
– Xenobiotic biodegradation and metabolism	102	1.69
Genetic information processing		
– Transcription	4	0.07
– Translation	81	1.34
– Folding, sorting and degradation	45	0.75
– Replication and repair	53	0.88
Environmental information processing		
– Membrane transport	285	4.73
– Signal transduction	93	1.54
Cellular processes		
– Cell growth and death	40	0.66
– Cell motility	55	0.91
– transport and catabolism	14	0.23
Total	2114	35.09

### Nitrogen fixation genes

One of the main characteristics of *E. alkalisoli* YIC4027 is its ability to fix nitrogen. The genome of *E. alkalisoli* YIC4027 contains 14 *nif* genes (*nifXNEKDH*, *nifSW*, *nifAB*, *nifZT*, and two copies of *nifQ*), 1 *fdx* gene (*fdxN*) and 11 *fix* genes (*fixNOQP*, *fixGHIS*, *fix*ABC). These genes are grouped into two clusters (Additional file 3: Figure S3). The first gene cluster contains *nifDKH* coding for the structural nitrogenase units, *nifQ*, *nifENX*, *nifB*, and *nifS* required for synthesis of the iron-molybdenum cofactor, *nifZT* and *nifW* genes responsible for nitrogenase maturation or catalytic stability [14] and *nifA* for transcription activation of *nif* genes [15]. The *fdxN* and *fixABC* genes present in this cluster are responsible for electron transfer to nitrogenase [16]. The second gene cluster includes the *fixNOQP* operon encoding a symbiotic cbb3-type heme-copper oxidase, and the *fixGHIS* operon encoding a membrane-bound protein complex required for formation of the cbb3-type heme-copper oxidase [17].

### Comparative analysis of the *E. alkalisoli* YIC4027 genome

**Fig. 2:**
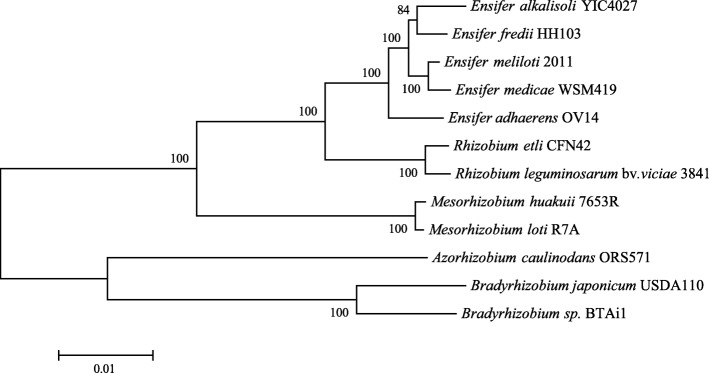
Phylogenetic relationships of *E. alkalisoli* YIC4027 and other rhizobia based on concatenated core protein sequences. The phylogenetic tree was constructed by the Maximum-Likelihood method using MEGA version 6.0 with 1000 bootstrap replicates

*E. alkalisoli* YIC4027 was proposed as a new species of *Ensifer* in our previous study [6]. To examine the relationship between YIC4027 and other rhizobia, we selected 11 completely sequenced genomes of representative rhizobial strains and constructed a phylogenetic tree based on their core genome. The obtained tree shows that YIC4027 is more closely related to *E. fredii* than to *E. meliloti* or *E. medicae* (Fig. 2).

**Fig. 3:**
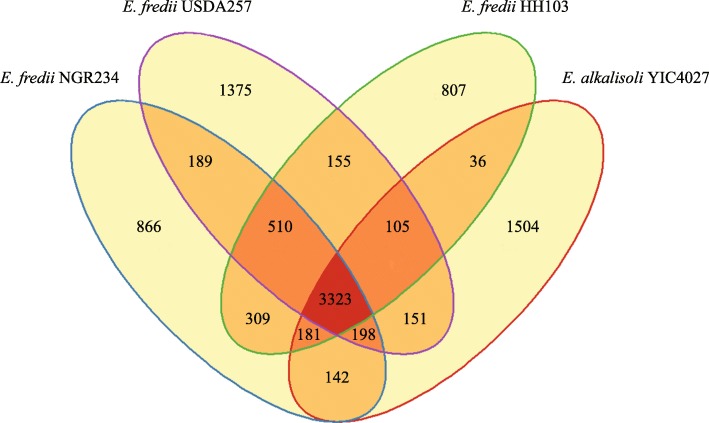
Venn diagram displaying overlaps and differences of orthologous genes in YIC4027 and *E. fredii* strains. Numbers represent the unique genes for an individual genome or common genes that are present in two or three genomes

The genome of *E. alkalisoli* YIC4027 was further compared to three closely related *E. fredii* strains at the protein level by analysis of orthologous genes (Fig. 3). Genome comparisons of these strains resulted in 9851 orthologous groups and 3323 of them were found to be conserved across the four genomes (representing 55.1% of the total number of YIC4027 genes). The number of genes unique to YIC4027 (1504 genes; 15.2%) was higher than that of *E. fredii* strains, i.e. USDA257 (1375, 13.96%), NGR234 (866, 8.7%) and HH103 (807, 8.1%). A pairwise comparison of YIC4027 with NGR234 resulted in 3844 orthologous genes, which is slightly higher than with USDA257 or HH103 (3777 and 3645 orthologs, respectively). These results indicate that YIC4027 is more closely related to NGR234 than to USDA257 or HH103. In order to further analyze differences in the genome structure between YIC4027 and the three *E. fredii* strains, synteny plots were performed to show the collinearity between their chromosomes. The results indicated that the chromosome of YIC4027 shows more synteny to those of NGR234 and HH103 than to that of USDA257 (Additional file 4: Figure S4). Furthermore, pYIC4027b displayed similarity to plasmid pSfHH103e of strain HH103 and a chromosome region of about 2 Mb in strain USDA257 [18].

### Nodulation factor biosynthesis genes

**Fig. 4:**
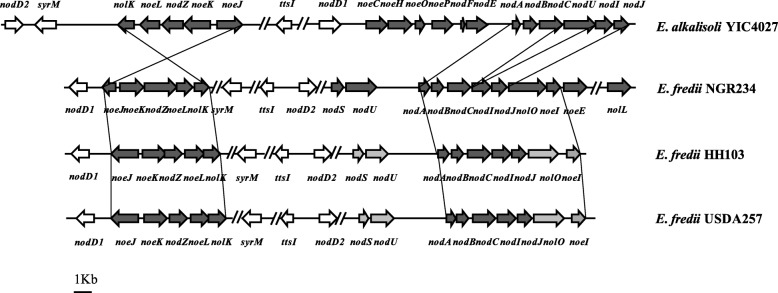
Arrangement of nodulation genes in *E. alkalisoli* YIC4027, *E. fredii* strains NGR234, HH103, and USDA257. Black arrows represent structural genes involved in NF synthesis. White arrows indicate genes involved in the transcriptional regulation of NFs. Gray arrows represent nonfunctional nodulation genes (truncated or mutated genes)

Although *E. alkalisoli* YIC4027 is closely related to *E. fredii* strains, their host ranges are remarkably different. *E. fredii* strains is able to nodulate more than 79 genera of legumes [10, 11], whereas *E. alkalisoli* YIC4027 is a specific symbiont of *S. cannabina* (based on nodulation tests performed so far). Rhizobial nodulation factors (NFs), surface polysaccharides, and secreted proteins are symbiotic determinants that play critical roles in nodulation of specific host plants [12, 19, 20]. NFs are a family of lipo-chitooligosaccharidic rhizobial signals with strain-specific substitution groups. These modifications may be required for bacterial recognition by specific NF receptors in host plants and subsequent nodule initiation. Hence, the chemical structure of NFs can determine host specificity [21]. Synthesis of NFs is governed by nodulation genes (i.e. *nod*, *nol*, and *noe*) [22]. Comparison of nodulation genes between *E. alkalisoli* YIC4027 and *E. fredii* strains revealed significant differences. As shown in Fig. 4, pYIC4027a harbors three gene clusters involved in NF production: (i) *nodABCUIJ*, (ii) *nolK*-*noeL*-*nodZ*-*noeK*-*noeJ*, and (iii) *nodEFnoeCHOP*. The identification of these genes suggests that YIC4027 produces NFs that are carbamoylated (*nodU*), fucosylated (*nolK*-*noeL*-*nodZ*-*noeK*-*noeJ*) and arabinosylated (*noeCHOP*). Furthermore, the presence of *nodEF* genes suggests that YIC4027 synthesizes NFs that possess unsaturated fatty acyl moieties. In contrast to YIC4027, only two NF synthesis gene clusters are present in the genomes of the three *E. fredii* strains: *nodABCUIJnolOnoeInoeE*, and *nolK*-*noeL*-*nodZ*-*noeK*-*noeJ* [18, 23, 24]. The organization and arrangement of nodulation genes of the three *E. fredii* strains are similar but the presence or functionality of the gene products may cause significant differences in NF structures. NFs of NGR234 are decorated with methyl-fucose, acetylated methyl-fucose, sulphated methyl-fucose as well as with carbamoyl and *N-*methyl groups [25]. In contrast, NFs of HH103 and USDA257are only fucosylated or methyl-fucosylated due to gene inactivation of *nolO*, *nodU*, *nodS* and absence of *nolL*, *noeE* [12, 26, 27]. As differences in *E. fredii* NFs may provide explanations for host specificity [12, 18], it is tempting to speculate that NF structures are responsible for the remarkably narrow host range of YIC4027. However, the precise biological roles of these differential genes are unclear and requires further experimental evidence.

### Genes related to polysaccharide production

In addition to NFs, various surface polysaccharides may function as determinants of host specific nodulation [21, 28]. Exopolysaccharide (EPS), lipopolysaccharide (LPS), capsular polysaccharide (KPS), and cyclic glucan (CG) have been implicated in infection thread formation and nodule formation [29, 30]. We predicted genes involved in the biosynthesis of these polysaccharides in *E. alkalisoli* YIC4027: the *exo*/*exs* gene cluster required for EPS production [31], the *greA* and *lpsBCDE* genes, which participate in LPS core biosynthesis [32], the *rkp-1*, *rkp-2* and *rkp-3* regions responsible for KPS production [33, 34], and *ndvBndvA*, which are involved in the synthesis and secretion of CGs [29, 35]. These gene clusters are also present in the genomes of the three examined *E. fredii* strains [23] (Additional file 5: Table S1). Overall, symbiotic polysaccharide synthesis genes were found to be well conserved in all four strains at the amino acid level. We therefore suggest that YIC4027 produces symbiotic surface polysaccharides that are similar to those of *E. fredii* strains.

### Genes involved in protein secretion

Protein secretion systems of rhizobia are also involved in host specificity, and can be divided into six types: type I to type VI [36]. The three analyzed *E. fredii* strains possess type I, II, III, and IV secretion systems. Remarkably, the genome of *E. alkalisoli* YIC4027 contains genes for type I, type III and type IV secretion systems, but no genes coding for a type II, type V and type VI secretion system being found (Table 4).

**Table 4:** Genes of *E. alkalisoli* YIC4027 related to protein secretion systems

System	Gene identification	Localization
Type I secretion		
*tolC*	EKH55_1288	Chromosome
*prtDE*	EKH55_2900 to 2901	Chromosome
*prsDE*	EKH55_5479 to 5480	pYIC4027b
Type III secretion (T3SS)		
T3SS-I		
*rhcC1 rhcC2 rhcJ rhcL rhcN rhcQRSTUV ttsI nopX*	EKH55_5609 to 5632	pYIC4027a
T3SS-II		
*rhcC1 rhcC2 rhcJ rhcL rhcN rhcQRSTUVZ*	EKH55_0998 to 1018	Chromosome
Type IV secretion (T4SS)		
T4SS-I		
*virB1-B11 virD4 virB1*	EKH55_5752 to 5930	pYIC4027a
T4SS-II		
*virB1-B11 virD4 virB2–3*	EKH55_4625 to 4740	pYIC4027b
Twin-arginine translocation (TAT) system		
*tatA, tatB, tatC*	EKH55_1307 to 1309	Chromosome
Secretion system		
*secB*	EKH55_3274	Chromosome
*secD*	EKH55_0177	Chromosome
*secE*	EKH55_1104	Chromosome
*secY*	EKH55_1142	Chromosome
*secG*	EKH55_1213	Chromosome
*secD/secF*	EKH55_1317	Chromosome
*secA*	EKH55_2587	Chromosome
*yajC*	EKH55_1316	Chromosome
*yidC*	EKH55_0075	Chromosome
*lebB*	EKH55_0711	Chromosome
SRP (signal recognition particle) components		
*ftsY*	EKH55_3169	Chromosome
*ffh*	EKH55_3175	Chromosome

The type II protein secretion system (T2SS) is encoded by a set of *gsp* (general secretory pathway) genes [37]. Proteins secreted by the T2SS must first be exported into the periplasmic space via the general secretion (Sec) or twin-arginine (Tat) pathways. The Tat systems of *Mesorhizobium loti* MAFF303099, *R. leguminosarum* bv. *viciae* 3841 and *R. leguminosarum* bv. *viciae* UPM791 were found to be required for effective nodulation of host plants [38–40]. The *gsp* genes are present in genomes of rhizobia that often possess a relatively broad host range, such as *E. fredii* NGR234, *E. fredii* HH103, *B. japonicum* USDA110, *M. loti* MAFF303099, *B. japonicum* BTAi1 and *Bradyrhizobium* sp. ORS278. In contrast, *gsp* genes were found to be absent in various narrow-host-range rhizobia such as *R. etli* CFN42, *R. leguminosarum* bv. *viciae* 3841, and strain *E. meliloti* 1021 [9, 13]. The genome of YIC4027 contains a set of genes encoding Sec or Tat pathways, but *gsp* genes were not found. Thus, we speculate that proteins secreted by the general secretory pathway are eventually implicated in host-specific nodulation.

**Fig. 5:**
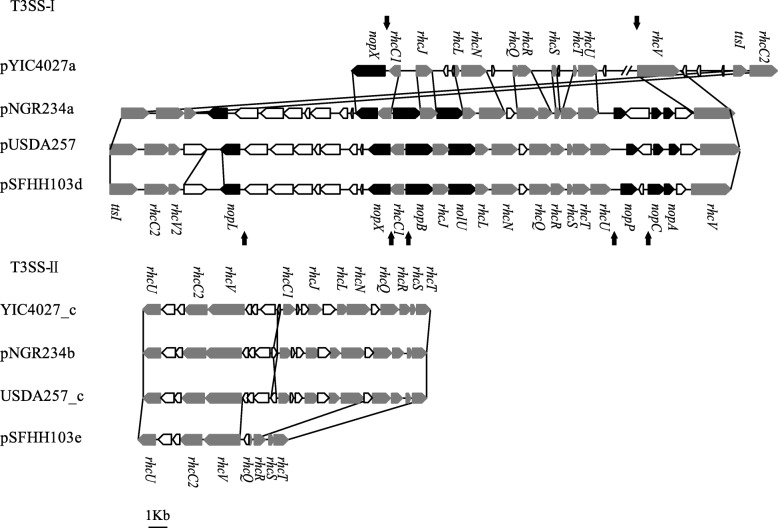
Comparison of the T3SS gene clusters in *E. alkalisoli* YIC4027 and *Ensifer fredii* strains NGR234, USDA257, and HH103. *Rhizobium* conserved (*rhc*) genes are shown in gray. Nodulation outer proteins (*nop*) genes are shaded in black. The positions of the *tts* boxes are indicated by vertical arrows

The rhizobial type III protein secretion system (T3SS) is involved in host-specific nodulation by delivering effector proteins through the lumen of a needle-like structure (pilus) into legume cells [19, 41, 42]. Two T3SS gene clusters (T3SS-I and T3SS-II) that match with those of *E. fredii* strains were identified in the genome of YIC4027. The T3SS-I cluster is located on the symbiotic plasmid pYIC4027a and the T3SS-II on the chromosome (Fig. 5 and Table 4).

The T3SS-I cluster of *Ensifer* strains contains (i) *Rhizobium* conserved (*rhc*) genes (involved in synthesis of a T3SS apparatus), (ii) nodulation outer protein (*nop*) genes (encoding secreted T3SS proteins, i.e. pilus proteins and effectors), and (iii) the *ttsI* gene encoding the transcriptional regulator TtsI. As in the three *E. fredii* strains, the *rhc* genes were also found in YIC4027 (Fig. 5). However, although present in the *E. fredii* genome, YIC4027 lacks the *nopABCLP* genes and only EKH55_5609 with 68% amino acid similarity to NopX (a putative translocon protein) was found in the T3SS-I cluster. The *nopA* of *E. fredii* strains encodes the pilus subunit protein NopA, which is required for a functional T3SS [43, 44]. The obvious lack of a *nopA* ortholog in the YIC4027 genome suggests that this strain does not possess a functional T3SS-I. Furthermore, a BLAST analysis indicated that homologous genes encoding T3SS effector proteins (NopBCDIJLMPT) of the three *E. fredii* strains are absent in the sequenced YIC4027 genome.

Type-III secretion (*tts*) boxes are conserved promoter motifs required for TtsI-dependent expression of rhizobial T3SS apparatus and effector genes [18, 45, 46]. To identify potential TtsI-regulated genes, we searched for *tts* boxes in the YIC4027 genome. The results indicated that two *tts* box-like elements are located in the T3SS-I cluster of YIC4027 (upstream of the *nopX* ortholog EKH55_5609 and EKH55_5627, a gene encoding a hypothetical protein) (Fig. 5 and Additional file 6: Table S2). In contrast, *nop* genes of *E. fredii* strains (such as *nopABCDIJLPTMX*) usually possess *tts* boxes in their promoters [18, 43, 45].

Studies have shown that the T3SS-I is involved in host-specific nodulation and that translocated effector proteins can modulate host defense reactions in *E. fredii* strains [47–50]. In contrast to the symbiotic role of the T3SS-I, no symbiotic defects were found for a T3SS-II mutant of NGR234 [9]. Therefore, it can be hypothesized that the presence of a T3SS-II of YIC4027 does not provide an explanation for the narrow host range of YIC4027. In conclusion, strain YIC4027 lacks *nopA* and known rhizobial effector genes, suggesting that strains lacking a functional T3SS tend to possess a narrow host range.

### Genes involved in adaptation to saline-alkaline soils

Since *E. alkalisoli* YIC4027 was isolated from a root nodule of *S. cannabina* grown in a saline-alkaline soil, its ability to grow well under saline (4% NaCl) and alkaline (pH 6–10) conditions corresponds to its environmental adaptation. The genome was inspected to search for genes which could account for adaptation to such environmental stress conditions. Uptake of potassium is a common response when rhizobia cope with osmotic stress [51]. Elevated K^+^ levels in response to osmotic stress act as a cellular signal for secondary responses [52]. Genes encoding three different types of K^+^ transporters, namely Kup, Trk, and Kdp, were found in YIC4027 (Additional file 7: Table S3). The *kup* gene is located on the chromosome of YIC4027, which encodes a constitutive K^+^ uptake system (Kup) with a modest affinity [53]. The *kdp* operon, located on the plasmid of YIC4027, encodes a high-affinity K^+^ uptake system (Kdp) which is functional even at low K^+^ concentrations [51]. The *trk* gene located on the chromosome and chromid of YIC4027, encodes the Trk system. This K^+^ uptake system, previously characterized in *E. meliloti* [51], is involved in K^+^ accumulation of osmotically stressed cells.

Glycine betaine and proline are effective osmoprotectants and their accumulation in bacteria is particularly important under high salt and osmotic stress conditions [54, 55]. The chromosome of YIC4027 contains genes required for biosynthesis of glycine betaine and proline (Additional file 7: Table S3). Furthermore, *proVWX* and *proP* genes were found on the chromosome of YIC4027. The *ProVWX* genes encode an ATP-Binding Cassette (ABC) transporter, which is predicted to possess a high affinity for glycine betaine [54]. The *ProP* gene, coding for L-proline transporter, contributes to osmotolerance in *Escherichia coli* and *Cronobacter sakazakii* [56, 57].

Trehalose is another important osmoprotectant that contributes to the growth of bacteria and plants under salt stress conditions [58, 59]. Five pathways of trehalose biosynthesis have been found in bacteria, namely the OtsA/OtsB, TreS, TreP, TreT and TreY/TreZ pathways [60]. On the chromid of YIC4027, genes coding for maltooligosyltrehalose synthase (*treY*, EKH55_4800), and maltooligosyltrehalose trehalohydrolase (*treZ*, EKH55_5127) were found. Trehalose synthesized by OtsA/OtsB is most widespread in bacteria, and often contributes to bacterial survival under stress conditions [61]. A trehalose-6-phosphate synthase (*otsA,* EKH55_5370), and a trehalose-6-phosphate phosphatase (*otsB,* EKH55_5369), were found to be present on the chromid of YIC4027.

Moreover, genes coding for proton antiporters contribute to osmoregulation and tolerance to saline-alkaline stress [62, 63]. The chromosome of YIC4027 contains *nhaABCDEFG* and *nhaP2*, a set of genes coding for a Na^+^/H^+^ antiporter and a K^+^/H^+^ antiporter, respectively. These antiporters allow for the bacteria to avoid excessive cation accumulation by importing H^+^ while simultaneously pumping out K^+^ and Na^+^ [62]. Homologous genes have been identified in genomes of salt- and alkali-tolerant rhizobacteria such as *Klebsiella* sp. D5A and *Enterobacter* sp. SA187 [54, 64].

### Genes involved in plant colonization

Chemotaxis and swimming motility contribute to rhizobial survival in the host rhizosphere and also to nodulation competitiveness, i.e. the nodulation efficiency of a given strain in the presence of other rhizobia [65–67]. The mobility of *E. alkalisoli* YIC4027 is ensured by their flagella (Additional file 8: Figure S5a). The genome analysis revealed that YIC4027 contains numerous motility-associated genes (Additional file 9: Table S4). Flagellar (*fla*, *flg*, *flh*, *fli*) and motility (*mot*) genes, located on the chromosome are required for the assembly of the flagellar apparatus. In addition, the genome harbors two gene clusters predicted to encode chemotaxis-like systems (Additional file 8: Figure S5b; Additional file 9: Table S4). Cluster 1 includes the genes encoding MCP, CheS, CheY, CheA1, CheW, CheR, CheB, and CheD proteins, and was located on the chromosome. Cluster 2 contains the genes encoding for CheR, CheW, MCP, CheA2, and CheB proteins, which was present on the chromid.

**Fig. 6:**
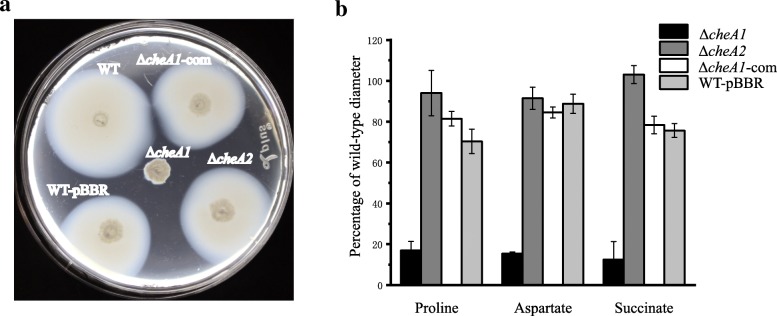
Chemotaxis behavior of *E. alkalisoli* YIC4027 and constructed *cheA* mutants. **a** A representative soft agar plate with proline as the sole carbon source. **b** The percentages of the chemotactic ring diameters of the mutants relative to those of the wild-type strain. Error bars represent standard deviations (SD) calculated from three independent experiments

**Fig. 7:**
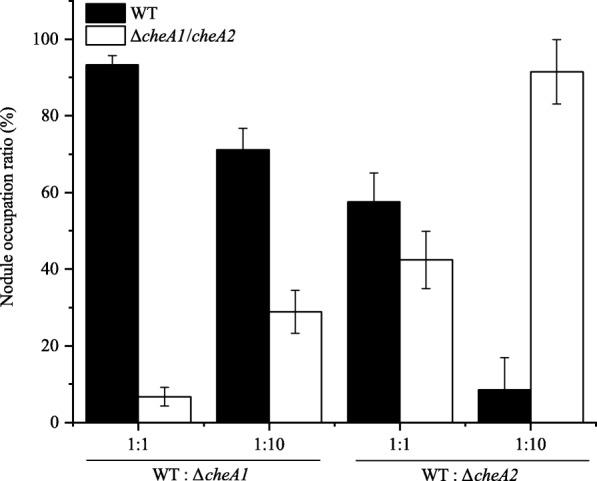
Nodulation efficiency of the constructed *cheA1* and *cheA2* mutants in competitive nodulation tests. *S. cannabina* seedlings were co-inoculated with the *E. alkalisoli* YIC4027 wild-type (WT) and an indicated mutant strain at ratios 1:1 and 1:10. Nodule occupancy was determined at the time of harvest. Error bars represent standard deviations calculated from at least three repetitions

To evaluate the role of chemotaxis in *E. alkalisoli* YIC4027, two *cheA* mutants (named Δ*cheA1* and Δ*cheA2*) were constructed and their chemotactic behavior was analyzed on soft agar plates with proline, aspartate, or succinate as carbon sources. The obtained results suggested that Δ*cheA1* was fully impaired in chemotaxis on soft agar plates, while Δ*cheA2* was not affected (Fig. 6a and b). The chemotaxis defects of the Δ*cheA1* mutant were restored by the introduction of a plasmid carrying the wild-type *cheA1* gene (Δ*cheA1*-com) (Fig. 6a and b). To analyze whether chemotaxis is related to nodulation competitiveness, *S. cannabina* roots were inoculated with the wild-type and *cheA* mutants alone or mixed in 1:1 and 1:10 ratios. When Δ*cheA1* and Δ*cheA2* were inoculated alone, the number and morphology of the nodules showed no differences as compared to the wild-type (data not shown). In competitive nodulation assays, however, the nodulation efficiency of Δ*cheA1* was significantly reduced in comparison to the wild-type strain. In contrast, the nodulation efficiencies of Δ*cheA2* and wild-type bacteria were similar in these experiments (Fig. 7). The growth kinetics of the wild-type and *cheA* mutants were similar (Additional file 10: Figure S6), excluding that the effect on chemotaxis and nodulation resulted from bacterial growth rates. In summary, these results showed that *cheA1* was essential for chemotaxis and nodulation competitiveness, while *cheA2* was considered to be dispensable.

## Discussion

*E. alkalisoli* YIC4027 is a motile rhizobium that efficiently fixes nitrogen in nodules of its host plant *S. cannabina* [6]. The complete genome sequence of YIC4027 provides the basis for a deeper understanding of molecular mechanisms underlying host specificity and environmental adaptations. The sequenced genome of YIC4027 allowed us to analyze its phylogenetic relationship with other rhizobia at a genomic level. We found that YIC4027 is closely related to various *E. fredii* strains. YIC4027 shares a conserved chromosomal backbone with *E. fredii* NGR234, HH103 and USDA257. A 2-Mb region of the USDA257 chromosome displays similarity with the megaplasmid of YIC4027. These results suggest that the megaplasmid of YIC4027 perhaps originated from an intragenomic transfer from its chromosome. Intragenomic transfer from the primary chromosome to a plasmid is an important evolutionary event that may have independently occurred in *Agrobacterium*, *Ensifer* and *Mesorhizobium* strains [13, 68].

Comparing the genomes of closely related strains with divergent host ranges is a promising approach for elucidating host range determinants [69]. In our study, three braod-host-range *E. fredii* strains served as a good reference to analyze the presence and absence of symbiosis-related genes in the YIC4027 genome. Our analysis suggest that the structure of YIC4027 NFs could play a role in host-specific nodulation. Remarkably, the YIC4027 genome harbors *noeCHOP*, suggesting that this strain produces arabinosylated NFs. In fact, arabinosylated NFs are not frequently produced by rhizobia but have been described for phylogentically different *Sesbania* microsymbionts [70, 71]. An *Azorhizobium caulinodans* ORS571 mutant deficient in production of arabinosylated NFs showed reduced nodule formation on the host plant *S. rostrata* [72]. We therefore suggest that arabinosylated NFs perceived by specific NF receptors play a crucial role in the association between YIC4027 and *S. cannabina.*

Furthermore, the lack of T2SS components and T3SS effectors in YIC4027 could provide explanations for the narrow host range of this strain. Previous studies have shown that NFs and T3SS effector proteins have a profound impact on host-specific nodulation, while the symbiotic role of the T2SS is not clear [13, 19, 73]. Further studies should be conducted to experimentally determine whether these factors play a role in host specificity.

Chemotaxis genes in rhizobia are required for efficient rhizosphere colonization and also can play a favorable role in nodulation competitiveness [65, 74]. We therefore analyzed YIC4027 genes related to chemotaxis. Our data demonstrated that YIC4027 possesses two chemotaxis clusters. The *che1* cluster was found to be located upstream of genes encoding for flagellar proteins. This cluster was homologous to the chemotaxis operon controlling flagellar motility in *E. meliloti*, *R. leguminosarum* bv. *viciae* and *A. tumefaciens* [65, 75, 76]. Mutation of the *cheA1* gene in YIC4027 resulted in impaired chemotaxis and reduced nodulation competitiveness, suggesting that the *cheA1* cluster plays a role in symbiosis-related motility and chemotaxis (Figs. 6 and 7). However, mutation of the *cheA2* gene did not obviously affect chemotaxis and nodulation efficiency in competition tests (Figs. 6 and 7). It is worth noticing that the response regulator gene *cheY* is absent in the *che2* cluster, suggesting that this cluster does not encode a complete chemotaxis signaling cascade. The gene organization of cluster 2 suggests that this cluster could possess alternative cellular functions [77]. Taken together, these results indicated that the *che1* cluster likely contributes to symbiosis-related rhizosphere colonization and nodulation competitiveness while the *che2* cluster may be considered dispensable for this process.

## Conclusions

The symbiotic association between nitrogen-fixing rhizobia and the legume *S. cannabina* is poorly understood [78]. *E. alkalisoli* YIC4027 is a predominant symbiont of *S. cannabina* growing in saline-alkaline soils of the YRD. In this work, we sequenced the complete genome of *E. alkalisoli* YIC4027 and compared it with *E. fredii* strains. Our results revealed differences with respect to NF synthesis genes and the lack of YIC4027 genes encoding T2SS components and T3SS effectors. In addition, the genome of YIC4027 contains various genes that may contribute to adaptation to saline-alkaline soils such as genes for glycine betaine synthesis, trehalose synthesis and proton antiporters. The genome of YIC4027 also harbors genes related to chemotaxis and the results of our mutant analysis indicated that the *che1* cluster plays a role in nodulation competitiveness*.* Finally, the genome of YIC4027 contains a high number of genes involved in metabolism and transport of amino acids and carbohydrates, suggesting that YIC4027 possesses highly efficient nutrient uptake systems which may provide competitive advantage in microbial rhizosphere communities [54, 79]. Altogether, the YIC4027 genome provides first insights into the molecular mechanisms underlying symbiosis and adaptation to saline-alkaline soils. Further research will be required to analyze the function of the identified genes in host-specific nodulation.

## Methods

### Bacteria and DNA preparation

*Ensifer alkalisoli* YIC4027 was cultured in tryptone-yeast extract (TY; 5 g/liter tryptone, 3 g/liter yeast extract, 0.6 g/liter CaCl_2_) medium for 2 days at 30 °C. A single colony was purified and its 16S rDNA sequence was verified before genomic DNA was prepared. High molecular weight genomic DNA was extracted by using an UltraClean® Microbial DNA Isolation Kit (Mobio laboratories, Carlsbad, USA). The DNA quantity and quality was checked by the Qubit assay on a Qubit fluorometer (Life Technologies, USA), and by measuring its absorbance at 260 nm and 280 nm using a Nanodrop Spectrophotometer (Thermo Scientific, UK).

### Genome sequencing, assembly and annotation

Genome sequencing of *E. alkalisoli* YIC4027 was performed using a Pacific Biosciences platform at the Berin Bio-technology Co., Ltd. (Shanghai, China). Genomic DNA was sheared with G-tubes (Covaris, Inc., USA), and fragments of 8-12 kb were isolated using AMPure beads (Beckman Coulter, USA). PacBio RS libraries were prepared with a DNA Template Prep Kit 2.0 (Pacific Biosciences, USA). The average PacBio RS library insert size (including adapters) was approximately 10 kb and samples were sequenced using PACBIO RSII.

The PacBio reads were assembled using the HGAP (Hierarchal Genome Assembly Processer) protocol. Glimmer 3.02 (http://ccb.jhu.edu/software/glimmer/index.shtml) and ZCURVE (https://omictools.com/zcurve-tool software) software were used to predict genes. RNAmmer [80] and tRNA-scan [81] were used to forecast the RNA and tRNA genes of the genome. BLASTP searches were conducted against the NCBI non-redundant (nr) protein database [82] and the Kyoto Encyclopedia of Genes and Genomes (KEGG) database [83] were performed for manual curation of the annotated genome. Clusters of Orthologous Groups (COG) annotation was carried out using RPS-BLAST against the CDD database [84].

### Phylogenetic analysis

A phylogenetic tree was constructed by the Maximum Likelihood (ML) method using concatenated core protein sequences from 12 representative rhizobial species (genera *Ensifer*, *Rhizobium*, *Mesorhizobium*, *Bradyrhizobium* and *Azorhizobium*). Clustal X2 was used to concatenate and align the protein sequences [85]. The final tree was generated using MEGA 6.0 [86] with a bootstrap value of 1000.

### Genome comparisons

A Venn diagram was constructed using GeneVenn [87] to compare the number of shared and unique genes based on clusters of orthologs. Genomic alignments were performed using ACT software [88]. The starting points of the replicons were adjusted to generate a clearer syntenic map.

### Identification of *tts* boxes in *E. alkalisoli* YIC4027

For identification of *tts* box sequences, the program fuzznuc of the EMBOSS package was used [89]. The intergenic regions of the YIC4027 genome were analyzed using the pattern “tcGTCAGcTT-tcGaaAGct” (capital letters indicate invariant nucleotides and the dash stands for any nucleotides). The search pattern was chosen based on known conserved *tts* box sequences in *E. fredii*, *B. japonicum*, and *M. loti* [18, 45, 46, 90].

### Electron microscopy

To observe flagella by transmission electron microscopy, YIC4027 cells were grown overnight with shaking at 30 °C in RB mannitol medium [91] to exponential phase to ensure motility. A droplet of the cell suspension was taken and adhered to Formvar-coated copper grids for 10 min. Excess amounts of bacteria were removed with a filter paper. Cells that adhered to the grids were stained with a drop of 1% phosphotungstic acid for 1 min. Examination was carried out with a transmission electron microscope JEM 1400 (Japan).

### Construction of mutant and complemented strains

For construction of a *cheA1* gene deletion mutant, a 606-bp upstream fragment (UF) from *E*. *alkalisoli* YIC4027 genomic DNA was amplified by PCR using the primer pair CheA1-UF and CheA1-UR, and a 579-bp downstream fragment (DF) was amplified using the primer pair CheA1-DF and CheA1-DR (for primers, see Additional file 11: Table S5). The upstream PCR product was then digested with KpnI-NdeI and inserted into the pCM351 plasmid [92], and the resulting plasmid was named as pCM351::UF. The downstream PCR product was digested with AgeI-SacI and cloned into pCM351::UF. The obtained plasmid pCM351::UF::DF was transformed into *E. coli* DH5α and checked by sequencing. Then the plasmid was transferred into *E*. *alkalisoli* YIC4027 by tri-parental conjugation using the helper plasmid pRK2013 [93]. The mutant candidates resistant to gentamicin were used for a PCR screen with the primer pair CheA1-UF and CheA1-DR. The obtained mutant was named Δ*cheA1*.

For construction of a *cheA2* mutant, a 543-bp upstream fragment (UF) was amplified by PCR using the primers CheA2-UF and CheA2-UR and a 651-bp downstream fragment (DF) with the primers CheA2-DF and CheA2-DR (Additional file 11: Table S5). The resulting upstream fragment was then digested with KpnI-NdeI, and coloned into the pCM351 plasmid. The plasmid was then digested with AgeI-SacI and ligated with the digested downstream fragment. The recombinant plasmid was introduced into *E*. *alkalisoli* YIC4027 for homologous recombination with the helper plasmid pRK2013. The *cheA2* mutant was then selected by the gentamicin resistance and was identified by PCR with the primers CheA2-UF and CheA2-DR.

For complementation of Δ*cheA1*, the coding sequences of *cheA1* and the upstream promoter region of the chemotaxis cluster were amplified by overlapping PCR. The amplicon was then cloned into the KpnI and XbaI sites of the broad-host-range vector pBBR1MCS-2 [94]. The DNA was verified by sequencing and the plasmid was introduced into the Δ*cheA1* mutant by triparental mating. Transformants were then recovered by selection for kanamycin resistance and verified by sequencing. The resulting strain was named Δ*cheA1*-com.

### Growth experiments

Strains were grown overnight in TY medium containing 25 μg ml^− 1^ nalidixic acid. Cultures were diluted with TY medium to adjust the optical density at 600 nm (OD_600_) to an initial value of 0.02. Cells were then grown on a rotary shaker (180 rpm) at 30 °C. Absorbance of the cultures at 600 nm was measured every 2 h. All data were depicted as means and standard deviations from three replicates.

### Chemotaxis assays

A soft agar plate assay was used to assess chemotaxis of *E*. *alkalisoli* YIC4027, as previously described [95]. The testing strains were cultured in RB medium containing 0.2% mannitol until exponential phase on a rotary shaker (180 rpm) at 30 °C. The cultures were then washed and resuspended in RB minimal medium to an OD_600_ of 1.0. Aliquots (5 μl) of the suspensions were inoculated onto RB minimal soft agar plates containing 10 mM carbon sources (proline, aspartate, and succinate) and 0.3% agar. Plates were then incubated for 3 to 5 days at 30 °C.

### Nodulation tests

Competitive nodulation tests were carried out with a few modifications as previously described [96]. Briefly, *S. cannabina* seeds were sterilized with concentrated sulfuric acid for 30 min and then germinated by incubation in the dark on inverted water-agar plates for 2 days at 30 °C. Wild-type and mutant bacteria were inoculated alone or mixed in 1:1 and 1:10 ratios into vermiculite-containing pots. Then the seedlings were planted into the pots and grown for 4–5 weeks in a greenhouse at 27 °C. To determine nodule occupancy, nodules were collected, surface-sterilized, and rinsed with sterilized water for five times. Then the nodules were crushed with a flame sealed sterilized pipette and plated on TY medium agar plates containing nalidixic acid. After 2 days of growth at 30 °C, the colonies were verified by PCR using the primer pairs CheA1-UF and CheA1-DR or CheA2-UF and CheA2-DR.

## Additional files

Additional file 1: **Figure S1.** COG categorization of CDSs in *E. alkalisoli* YIC4027. (TIF 1202 kb)

Additional file 2: **Figure S2.** KEGG classification of CDSs in *E. alkalisoli* YIC4027. (TIF 642 kb)

Additional file 3: **Figure S3.** Nitrogen fixation gene clusters of *E. alkalisoli* YIC4027. (EPS 663 kb)

Additional file 4: **Figure S4.** Genomic alignments of *E. alkalisoli* YIC4027 with three *E. fredii* strains. **a)** Chromosome alignments of *E. alkalisoli* YIC4027 and *E. fredii* NGR234. **b)** Chromosome alignments of *E. alkalisoli* YIC4027 and *E. fredii* HH103. **c)** Alignments of the *E. fredii* USDA257 chromosome with the *E. alkalisoli* YIC4027 chromosome (top) and the plasmid pYIC4027b (bottom). Red and blue lines indicate syntenic matches in the forward and reverse orientations, respectively. Numbers represent the position of base pairs on the respective replicons. (EPS 16923 kb)

Additional file 5: **Table S1.** Similarities (%) between surface polysaccharide biosynthesis genes of *E. alkalisoli* YIC4027 and three *E. fredii* strains. (XLS 288 kb)

Additional file 6: **Table S2.** Identified *tts* box sequences of *E. alkalisoli* YIC4027. Selected *tts* box sequences of *E. fredii* strains (NGR234, USDA257 and HH103) are shown for comparison. (XLSX 11 kb)

Additional file 7: **Table S3.** Genes involved in salt-alkali tolerance in *E. alkalisoli* YIC4027. (XLS 273 kb)

Additional file 8: **Figure S5.** An electron micrograph showing flagella and chemotaxis gene clusters of *E. alkalisoli* YIC4027. **a)** Transmission electron microscopy of a YIC4027 bacterium with flagella. Cells were negatively stained with phosphotungstic acid. Bar, 1 μm. **b)** Organization of chemotaxis gene clusters in the YIC4027 genome. The *cheA1* and *cheA2* genes are shown in red. (EPS 5867 kb)

Additional file 9: **Table S4.** Genes involved in chemotaxis, flagellation formation, and motility of *E. alkalisoli* YIC4027. (XLS 272 kb)

Additional file 10: **Figure S6.** Growth of *E. alkalisoli* YIC4027 wild-type (WT) and *cheA* mutants in TY medium. (EPS 839 kb)

Additional file 11: **Table S5.** Primers used in this study. (XLS 20 kb)

## Data Availability

The complete genome sequence of *E*. *alkalisoli* YIC4027 has been submitted to GenBank under the accession number CP034909-CP034911.

## Abbreviations

ANI: Average nucleotide identity
CDS: Coding sequences
CG: Cyclic glucans
COG: Clusters of orthologous groups
EPS: Exopolysaccharide
gsp: general secretory pathway
KEGG: Kyoto encyclopedia of genes and genomes
KPS: Capsular polysaccharide
LPS: Lipopolysaccharide
NFs: Nodulation factors
T2SS: type II secretion system
T3SS: type III protein secretion system
TAT: Twin-arginine translocation system
YRD: Yellow river delta
