# Supplementary figures and images for: The genome of Ensifer alkalisoli YIC4027 provides insights for host specificity and environmental adaptations
Source: BMC Genomics. 2019 Aug 12;20:643. doi: 10.1186/s12864-019-6004-7 (PMC6689892; doi:10.1186/s12864-019-6004-7)

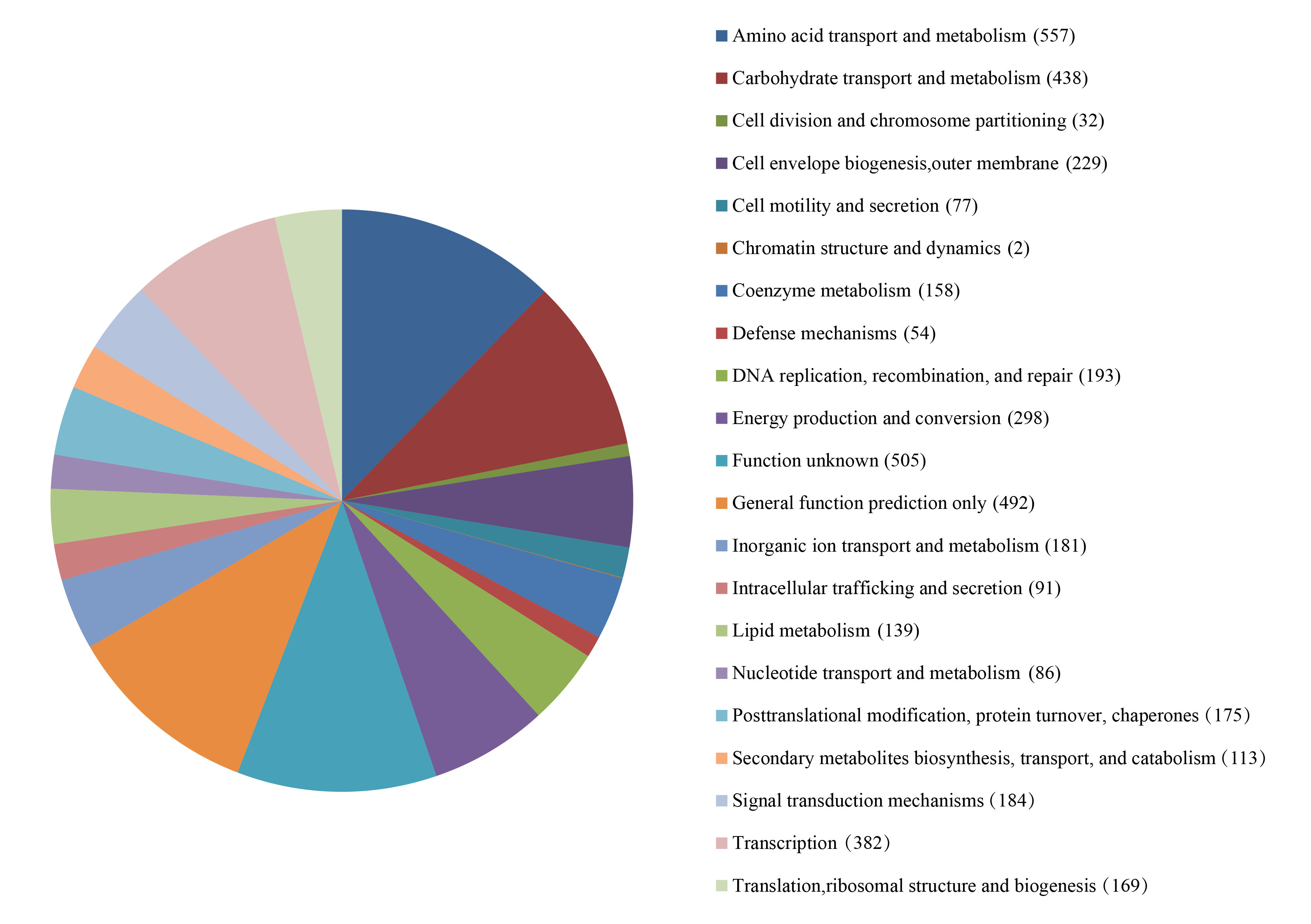

Supplement: Supplementary file 1 — Figure S1. COG categorization of CDSs in E. alkalisoli YIC4027. (TIF 1202 kb) [file 12864_2019_6004_MOESM1_ESM.tif]

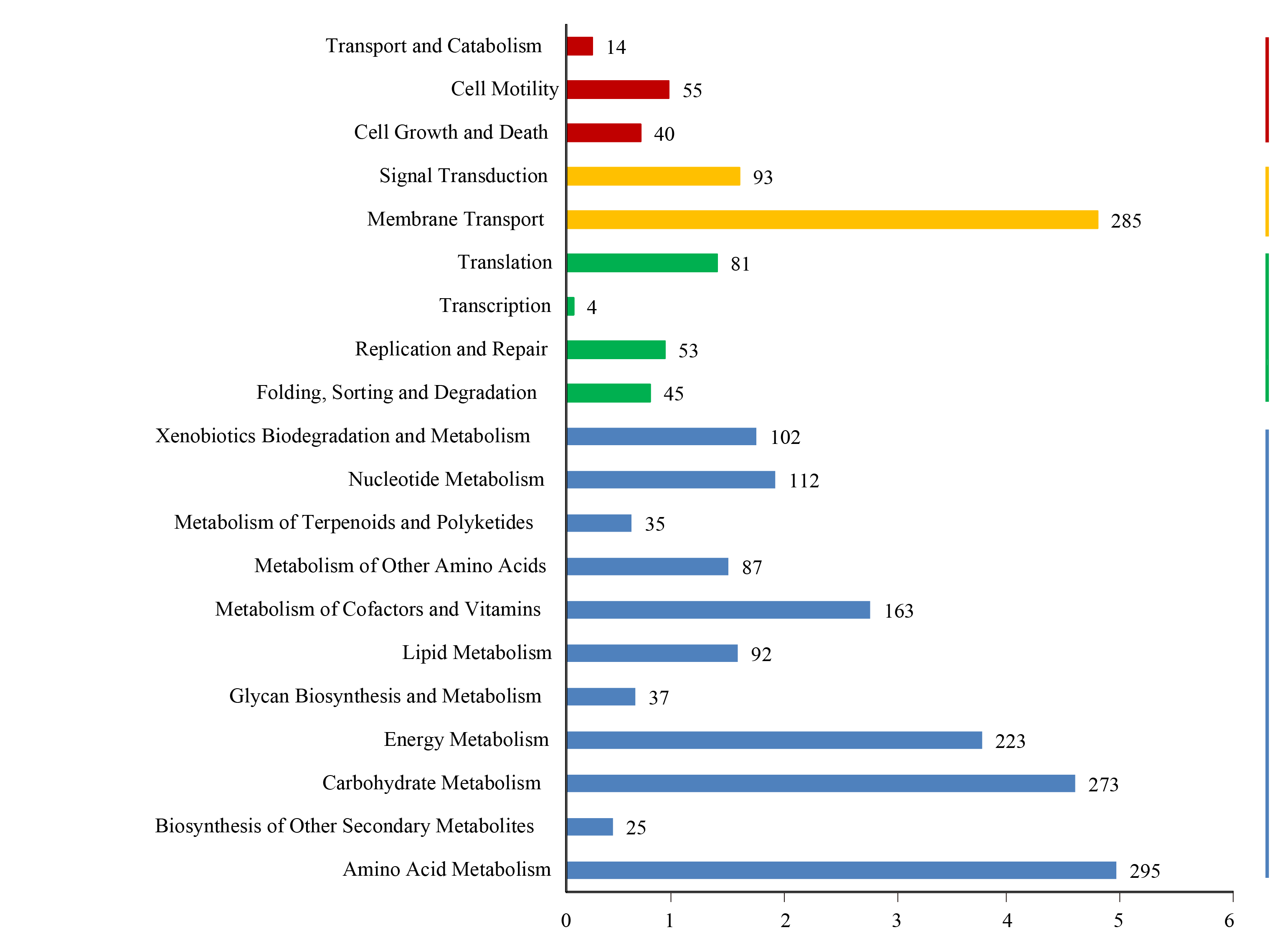

Supplement: Supplementary file 2 — Figure S2. KEGG classification of CDSs in E. alkalisoli YIC4027. (TIF 642 kb) [file 12864_2019_6004_MOESM2_ESM.tif]
